# Weight loss interventions for improving fertility: a synthesis of current evidence

**Published:** 2024-03-01

**Authors:** Emma Schneider, Oliver Hamer, Chris Smith, James Hill

**Affiliations:** Liverpool University Hospitals NHS Foundation Trust; University of Central Lancashire; University of Central Lancashire; University of Central Lancashire

## Abstract

Infertility is a widespread issue which is estimated to affect up to 17.5% of the global population. Evidence suggests that the most common causes of female infertility are ovulation disorders (e.g., polycystic ovary syndrome). That said, lifestyle factors such as dietary patterns, stress, alcohol consumption, smoking, and obesity are key determinants which have been shown to impact female physiology and significantly decrease the chances of conception. Obesity has been widely recognized as a significant factor that negatively impacts ovarian stimulation in women and is associated with several reproductive disorders, including anovulation, subfertility, and infertility. Despite improvements in fertility treatments, obesity remains a challenge particularly for fertility clinics because of the poorer pregnancy outcomes observed within the population. In this article, we will explore the effects of weight loss on female fertility and review the various strategies that have been shown to be effective in reducing obesity and improving reproductive outcomes.

## Introduction

Infertility, which refers to the inability to conceive or maintain a pregnancy to the point of a live birth, is a widespread issue which is estimated to affect up to 17.5%of the global population ^1–5^. According to the World Health Organization, infertility has become a major public health concern, with increasing prevalence over the last 20 years ^2^. Globally, the age-standardized prevalence rate of female infertility has increased by 15% from 1990 to 2017 (1571.35 per 100,000), with the highest rates in those aged 35–39 ^1,2^. The aetiology of infertility is complex and includes factors such as genetic mutations, ovulatory disorders, endometriosis, unexplained infertility and lifestyle factors ^6–8^. Evidence suggests that the most common causes of female infertility are ovulation disorders (e.g., polycystic ovary syndrome) ^9^. That said, lifestyle factors such as dietary patterns, stress, alcohol consumption, smoking, and obesity are key determinants which have been shown to impact female physiology and significantly decrease the chances of conception ^6–8^.

Obesity has been widely recognized as a significant factor that negatively impacts ovarian stimulation in women and is associated with several reproductive disorders, including anovulation, subfertility, and infertility ^10,11^. Research has highlighted the risk of heighted BMI, suggesting that with each unit increase in BMI (>30kg/m^2^), ovulatory function could decrease by up to 5% ^12^. Women who are obese often experience reduced spontaneous ovulation and are more likely to develop polycystic ovary syndrome (PCOS) ^10^. Polycystic ovary syndrome can also lead to weight gain and is a key concern for patients as it often leads to decreased efficacy and reduced success of assisted reproductive technology (e.g., in vitro fertilisation, (IVF)) ^10^. Despite improvements in fertility treatments, obesity remains a challenge particularly for fertility clinics because of the poorer pregnancy outcomes observed within the population ^13^.

In recent years, weight loss has emerged as a key strategy for improving fertility and assisted reproductive technology outcomes in adults with obesity ^13,14^. A study has found that weight loss of 5% and lower BMI (with normal triglyceride level), are independent predictors of pregnancy in women seeking fertility treatment ^13^. To achieve weight loss, approaches such as diet and exercise, have been identified as key strategies shown to improve reproductive outcomes (in both men and women) ^15^. However, there are a wide variation of diet, exercise, pharmacological, surgical, and psychological interventions that promote weight loss which often creates uncertainty among patient as to which is most effective ^16^. This article examines the impact of weight loss on female fertility, discussing effective dietary and physical activity strategies for reducing obesity and improving reproductive outcomes. It critically evaluates and expands upon the findings of a systematic review by Hunter et al. (2021), which assessed the effectiveness of non-pharmacological interventions for weight loss in individuals experiencing infertility.

## Methods of Hunter et al. (2021)

This review was an update of a previous review. This review undertook a comprehensive multi-database updated search from March 2016 until March 2020. Additional citation screening of all included studies was undertaken. Only randomized controlled trials (RCTs) involving individuals who were overweight (BMI = ≥ 25 kg/m^2^), actively attempting pregnancy, experiencing infertility and without medical conditions that could cause weight fluctuations receiving type of lifestyle intervention aimed at changing weight were included. Screening, data extraction and assessment of bias (Cochrane risk-of-bias-tool) was undertaken by two reviewers independently. Effects were estimated using a random effects model, employing relative risk (RR) for dichotomous outcomes and mean differences (MD) for continuous variables. The authors assessed publication bias by visually inspecting the funnel plot for outcomes where there were 10 or more studies.

## Results of Hunter et al. (2021)

With this update eight additional studies were identified resulting in fifteen RCTS in total being included in the review. A risk of bias assessment was undertaken, which demonstrated variability in the quality of studies. Out of the 15 analysed RCTs, all studies had at least one high and one unclear risk of bias in two domains. Due to the small number of studies included in this review, interpretation of the funnel plots was limited. However, no observation of asymmetry was observed for live birth rate, pregnancy rate and changing weight outcomes.

### Primary outcomes

The review found that overall participants randomized to receive lifestyle interventions achieved greater reduction in weight (MD= -5.24 kg, 95% CI -7.14 to -3.35) compared to those in the control groups. Patients who received diet and exercise interventions experienced a significant decrease in weight compared to those who received no or minimal intervention (MD = -4.66 kg, 95% CI -6.03 to −3.30) and immediate access to assisted reproductive technology (MD = -4.16 kg, 95% CI -6.87 to −1.44). Single study evidence indicates that very low-calorie diet plus exercise may be more effective than standard diet and exercise (MD = -9.00 kg, 95% CI −15.50 to -2.50). There is also single study evidence that diet only interventions may reduce weight compared to those who received no or minimal intervention (MD = -5.23 kg, 95% CI -7.42 to -3.04) and immediate access to assisted reproductive technology (MD = -10.29 kg, 95% CI -11.42, -9.16). There was no evidence of effect when comparing exercise only to no or minimal intervention and diet and exercise interventions compared to diet interventions only. In all meta-analyses examining the effects of weight reduction, there was either nonsignificant or moderate heterogeneity observed.

Overall patients receiving lifestyle interventions had an increased risk of a live birth (RR=1.46, 95% CI 1.04 to 2.04) compared to those in control groups. Diet and exercise statistically significantly increased the risk of live birth compared to no or minimal intervention (RR = 2.20, 95% CI 1.23 to 3.94). However, there was no evidence of difference when compared to immediate access to assisted reproductive technology. There was no evidence of effect when comparing very low-calorie diet compared to standard diet and exercise and diet alone compared to no or minimal intervention or immediate access to assisted reproductive technology. In all meta-analyses examining the effects of risk of a live birth, there was either nonsignificant or substantial heterogeneity observed.

Reduced calorie diet and exercise increased clinical pregnancy rate compared to no or minimal intervention (RR = 1.87, 95% CI 1.20 to 2.93) but not compared to immediate access to assisted reproductive technology (RR = 1.43, 95% CI 0.83 to 2.48). Single study evidence suggested that there was no evidence of difference between very low-calorie diet and standard diet and exercise compared against all comparisons.

### Secondary outcomes

Out of the 23 secondary comparisons only five were statistically significant (see Table 1 for full results of secondary outcomes). There was a statistically significant improvement in ovulation (RR = 4.24, 95% CI 1.45 to 12.39) and menstrual cycle irregularity (RR = 3.67, 95% CI 1.13 to 11.92) when comparing diet and exercise to no/minimal intervention. There was also a statistically significant improvement in natural conception rates (RR = 3.92, 95% CI 1.34 to 11.48) and conception rates following assisted reproductive technology (RR = 3.92, 95% CI 1.35 to11.48) when comparing diet alone to immediate access to assisted reproductive technology. Finally, there was a statistically significant improvement in number of oocytes retrieved when comparing diet alone to no or minimal intervention (MD = -3.57, 95% CI -6.87 to -0.27).

## Commentary

The quality of a systematic review’s methods can be evaluated using the Amstar-2 tool, which identifies whether 16 crucial methodological processes have been carried out ^17^. In the critical appraisal of this review, 10 out of the 16 criteria were deemed satisfactory using the Amstar2 tool. This systematic review had several areas of concern. Firstly, it did not provide a clear explanation of the study design inclusion criteria. Where it is indicated in the aims that additional RCTs will be identified this isn’t specifically stated as an inclusion criterion. Additionally, the review did not provide a list of excluded studies with justification for their exclusion. Another significant issue is that the authors did not report on the sources of funding for the studies included, which raises potential concerns about conflicts of interest of included studies. The review also failed to report on the potential impact of the risk of bias in individual studies on the results of single outcomes in the meta-analysis. Finally, the authors did not account for the risk of bias in individual studies when interpreting the results of the review. The methodological issues in the review and the included studies suggest that moderate caution is required when interpreting the findings from this review.

## Implications for Clinical Practice

This systematic review found that diet and exercise may help to reduce weight for individuals who were overweight (BMI = ≥ 25 kg/m^2^) and actively attempting pregnancy. When interpreting these findings, it is difficult to establish the clinical significance of the mean estimate of weight reduction presented in the review as no specific time period was given for time of outcome. Furthermore, these estimates may be less or more impactful depending on the starting weight of the participants. When interpreting the external validity of this review it is important to be aware that none of the studies were carried out in the UK and the BMI threshold was ≥ 25 kg/m^2^ for the intervention programs.

There was some evidence that diet and exercise may increase the risk of live birth and clinical pregnancy. With less certainty, there was some evidence that diet and exercise may improve ovulation and reduce menstrual cycle irregularity. The positive impact observed in this review regarding the effects of diet and exercise aligns with the guidelines provided by the National Institute for Health and Care Excellence (NICE) for addressing fertility problems (Overview | Fertility problems: assessment and treatment | Guidance | NICE). These guidelines recommend that women with a BMI of 30 or above, who are not ovulating, should be informed that losing weight is likely to increase their chances of conceiving.

Regarding diet only, there was some evidence to suggest that diet only may help weight loss. Furthermore, there was also tentative evidence that it could help improve conception rates. However, according to the NICE guidelines from 2013, women should be informed that participating in a group program that involves exercise and dietary advice leads to higher pregnancy rates compared to receiving weight loss advice alone (Overview | Fertility problems: assessment and treatment | Guidance | NICE). Although, in the systematic review, although both group-based and individualized interventions were included, they were not directly compared. While both group-based and individualized interventions were included in the systematic review, there was no direct comparison between them. One study within the review suggested that group meetings in their research led to improved retention, but this finding was not supported by other studies included in the review. Overall based upon the review’s findings and NICE guidelines both exercise and diet may provide the optimum approach.

However research suggests that professionals in primary care rarely implement guidelines around weight (https://bmjopen.bmj.com/content/5/1/e006642) with one study showing that fewer than half of people living with obesity in the UK (47%) have had a discussion with a health care professional about their weight in the past 5 years, despite these health care professionals being the key to accessing further specialist weight management support (Changing the narrative around obesity in the UK: a survey of people with obesity and healthcare professionals from the ACTION-IO study | BMJ Open) therefore it may be unlikely that this conversation regarding weight loss and fertility would routinely happen.

Interventions including Very Low Calorie Diets (VLCDs) showed the greatest reduction in weight, although at the moment there is insufficient research to fully determine the safety and acceptability of this in the pre-conception period ^18^. NICE only recommend considering VVLCDs as part of a multi-component intervention for those who have a clinically assessed need to rapidly lose weight (e.g. seeking fertility services) (Recommendations | Obesity: identification, assessment and management | Guidance | NICE).

Sub-analysis by starting BMI was not able to be completed due to insufficient studies within each category. It is likely that starting BMI would be an important determinant impacting the outcomes of the various interventions. The review also looked at lifestyle interventions only, rather than comparing these to combined lifestyle and pharmacological or surgical interventions, which have also been shown to result in weight loss to improve fertility ^19,20^.

## Recommendations for Future Research

The review was not able to identify any RCTs looking at the effects of weight loss on fertility outcomes on men and therefore future research exploring the role of male BMI and weight loss in fertility is required. The review also suggested considering couple-based approaches to weight loss within the fertility seeking context may be feasible and acceptable.

The review also did not include lifestyle interventions in combination with pharmacology and/or surgery which may produce improved fertility outcomes compared to lifestyle interventions alone, due to the greater weight loss achieved. The review did not assess the potential harms of the interventions considered, which is an important consideration in evaluating the overall impact of the interventions.

## Practise challenge questions

1. What are the limitations and strengths of the evidence synthesised by the systematic review?
2. What are the limitations of recommending Very Low-Calorie Diets (VLCDs) to promote weight loss for improving fertility?
3. What intervention(s) lead to higher pregnancy rates compared to receiving weight loss advice alone?

## Figures and Tables

**Table 1:** Secondary outcomes full results

Outcome	Intervention	Control	Effect (95% confidence interval)	I^2^ =, P=
Improvement in ovulation				
Improvement in ovulation	Diet and exercise	No or minimal intervention	RR = 4.24 (95% CI 1.45, 12.39)	0%, 0.80
Improvement in ovulation	Very low-calorie diet and exercise	Standard diet and exercise	RR = 7.00 (95% CI 0.43, 114.70)	N/A
Improvement in ovulation	Pedometer, diet and exercise counselling	Diet and exercise counselling	RR = 4.40 (95% CI 0.59, 33.07).	N/A
Menstrual cycle irregularity				
Menstrual cycle irregularity	Diet and exercise	No or minimal intervention	RR = 3.67 (95% CI 1.13, 11.92)	N/A
Natural conception rates				
Natural conception rates	Lifestyle interventions (all intervention types)	All control types	RR = 2.25 (95% CI 1.42, 3.59)	N/A
Natural conception rates	Diet only	Immediate access to assisted reproductive technology	RR = 3.92 (95% CI 1.34, 11.48).	N/A
Natural conception rates	Diet and exercise	Immediate access to assisted reproductive technology	RR = 2.20 (95% CI 0.98, 4.93)	62%, 0.11
Natural conception rates	Diet only	No or minimal intervention	RR = 4.52 (95% CI 0.23, 88.28)	N/A
Natural conception rates	Exercise only	No or minimal intervention	RR = 2.25 (95% CI 0.25, 20.38)	N/A
Conception rates following assisted reproductive technology				
Conception rates following assisted reproductive technology	Diet and exercise	No or minimal intervention	RR = 4.52 (95% CI 0.23, 88.38)	N/A
Conception rates following assisted reproductive technology	Diet alone	Immediate access to assisted reproductive technology	RR = 2.20 (95% CI 0.98, 4.93)	N/A
Conception rates following assisted reproductive technology	Diet alone	Immediate access to assisted reproductive technology	RR = 3.92 (95% CI 1.35,11.48)	N/A
Conception rates following assisted reproductive technology	Exercise alone	No or minimal intervention	RR = 2.25 (95% CI 0.25, 20.38)	N/A
Number of oocytes retrieved				
Switching	Diet alone	No or minimal intervention	MD = -3.57 (95% CI -6.87, -0.27)	N/A
Number of oocytes retrieved	Diet alone	Immediate access to assisted reproductive technology	MD = -0.44 (95% CI -1.69, 0.81)	N/A
Miscarriage rates per participant				
Miscarriage rates per participant	Diet and exercise	No or minimal intervention	RR = 0.95 (95% CI 0.79, 1.15)	66%, 0.09
Miscarriage rates per participant	Diet and exercise	Immediate access to assisted reproductive technology	RR = 0.95 (95% CI 0.90, 1.01)	0%, 0.42
Miscarriage rates per participant	Diet alone	Immediate access to assisted reproductive technology	RR = 0.98 (95% CI 0.94, 1.03)	N/A
Miscarriage rates per pregnancy				
Miscarriage rates per pregnancy	Diet alone	Immediate access to assisted reproductive technology	RR = 0.96 (95% CI 0.85, 1.09)	N/A
Miscarriage rates per pregnancy	Diet and exercise	No or minimal intervention	RR = 1.22 (95% CI 0.75, 1.99)	N/A
Miscarriage rates per pregnancy	Diet and exercise	Immediate access to assisted reproductive technology	RR = 0.92 (95% CI 0.82, 1.03).	N/A
Miscarriage rates per pregnancy	Diet and exercise	Immediate access to assisted reproductive technology	RR = 0.90 (95% CI 0.82, 0.99)	N/A
Time to conception				
Miscarriage rates per pregnancy	Diet and exercise	Immediate access to assisted reproductive technology	Median = 7.2 months (quartile ranges 2.6,12.0)	N/A

## Key findings

- Patients who receive diet and exercise interventions experienced a significant decrease in weight compared to those who received no or minimal intervention
- There was no evidence of effect when comparing exercise only to no or minimal intervention and diet and exercise interventions compared to diet interventions only.
- Further research is needed to assess if there are any potential harms of diet and physical activity interventions as a means of achieving weight loss.
